# Many Ways to Communicate—Crosstalk between the HBV-Infected Cell and Its Environment

**DOI:** 10.3390/pathogens12010029

**Published:** 2022-12-24

**Authors:** Annika Jasmin Walter, Maarten A. van de Klundert, Stephanie Jung

**Affiliations:** 1 Institute of Cardiovascular Immunology, University Hospital Bonn, University of Bonn, 53127 Bonn, Germany; 2 Division of Infectious Diseases, Department of Medicine, Karolinska Institutet, 14152 Stockholm, Sweden

**Keywords:** hepatitis B virus, cell-cell interactions, hepatocellular carcinoma, innate and adaptive immune response, extracellular vesicles, viral spread

## Abstract

Chronic infection with the hepatitis B virus (HBV) affects an estimated 257 million people worldwide and can lead to liver diseases such as cirrhosis and liver cancer. Viral replication is generally considered not to be cytopathic, and although some HBV proteins may have direct carcinogenic effects, the majority of HBV infection-related disease is related to chronic inflammation resulting from disrupted antiviral responses and aberrant innate immune reactions. Like all cells, healthy and HBV-infected cells communicate with each other, as well as with other cell types, such as innate and adaptive immune cells. They do so by both interacting directly and by secreting factors into their environment. Such factors may be small molecules, such as metabolites, single viral proteins or host proteins, but can also be more complex, such as virions, protein complexes, and extracellular vesicles. The latter are small, membrane-enclosed vesicles that are exchanged between cells, and have recently gained a lot of attention for their potential to mediate complex communication and their potential for therapeutic repurposing. Here, we review how HBV infection affects the communication between HBV-infected cells and cells in their environment. We discuss the impact of these interactions on viral persistence in chronic infection, as well as their relation to HBV infection-related pathology.

## 1. Introduction

Worldwide, more than 296 million people are infected with hepatitis B virus (HBV), and each year an estimated 820,000 people die as a consequence. Most of these deaths are due to chronic HBV infections (CHB), in which continuing viral replication and the resulting inflammation lead to severe liver damage and liver cancer, which occurs in about 25% of the chronically infected individuals [1]. It is poorly understood why the immune system fails to clear the infection. Antiviral responses do develop, but the cells that can recognize HBV do not function properly [2,3]. This leads to a “status quo”, in which HBV specific immune cells suppress viral replication but fail to clear the infection [4]. In resource-rich settings, HBV infection can be treated with nucleoside or nucleotide analogues (NAs), which prevent viral replication but do not affect the stable HBV DNA in already-infected hepatocytes. Due to the natural turnover of hepatocytes, the number of infected cells becomes less and less, and after years of therapy, often no markers of HBV replication can be found in the serum anymore. However, small amounts of HBV genomic DNA persist in liver parenchymal cells, and if therapy is discontinued, the viral infection is re-established from this DNA. Therefore, to reduce the chances of developing HBV infection-related pathology, NAs have to be taken lifelong. A safe and effective vaccine that can protect children right after birth is available; however, this strategy cannot prevent all perinatal transmissions, especially those occurring before or during delivery, and does not suffice as a means to contain the current epidemic [5]. Although in some countries, the introduction of perinatal HBV vaccination as standard care has greatly reduced HBV incidence, the number of HBV cases is increasing worldwide. Indeed, whereas the incidence of—and the mortality due to—other infections such as human immunodeficiency virus (HIV) and tuberculosis (TB) are declining, the incidence of—and mortality due to—viral hepatitis, mainly HBV, are rising. The WHO has urged scientists and policy makers worldwide to reckon these treat-and-device strategies to combat HBV.

If not treated, HBV infection can lead to liver fibrosis, liver cirrhosis, and finally to liver failure. Most HBV-related deaths, however, are attributable to a specific type of liver cancer: hepatocellular carcinoma (HCC), which develops in about 10–25% of HBV-infected individuals [6,7]. The odds of developing HBV infection-related HCC differ for the different HBV genoypes [8]. The median survival of untreated HCC is 8 months. The 5-year survival rate of HCC patients is about 14% in the US, and is lower in developing countries where HCC is more common [9]. Due to the high mortality rate, HCC is the fourth leading cause of cancer-related death worldwide, and the vast majority of these cancers are caused by HBV infection. 

Many aspects of HBV infection contribute to the development of HBV-related HCC. The ongoing immune responses cause liver inflammation and dysregulate various processes. Importantly, in most HBV infection-related HCCs, HBV DNA can be found integrated in the host genome. Such integrations may contribute to HCC by affecting the expression of oncogenes near the integration site [10], but also the expression of viral proteins may significantly contribute to the development of HBV-related HCC [11,12]. Thus, the development of HBV-related HCC is a multifactorial process to which several different aspects of HBV replication may contribute. 

**Figure 1:**
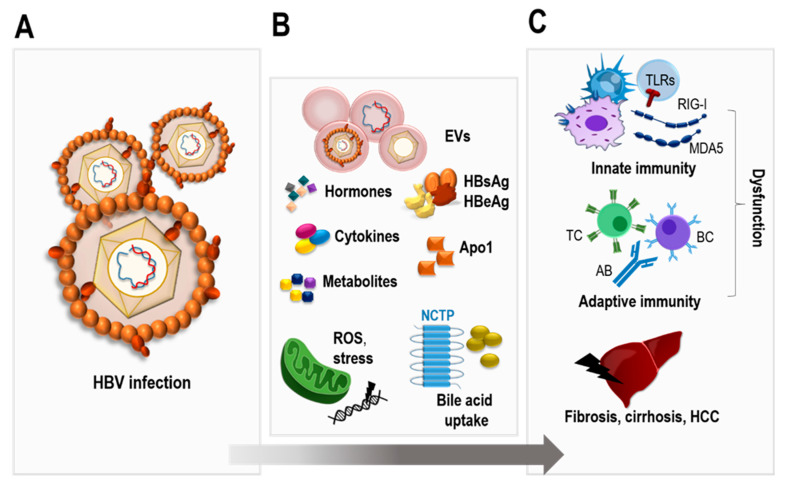
Overview on HBV-influenced factors. In hepatitis B virus (HBV) infections (**A**), various mediators (**B**) are associated with HBV-induced immune dysfunction and liver damage (**C**). EVs: extracellular vesicles (EVs); HBsAg: hepatitis B surface antigen; HBeAg: hepatitis B early antigen; ApoA1: apolipoprotein A1; ROS: reactive oxygen species; NTCP: sodium taurocholate cotransporting polypeptide; TLRs: toll-like receptors; RIG-I: retinoic acid inducible gene I; MDA5: melanoma differentiation-associated protein 5; TC: T cells; BC: B cells; AB: antibodies; HCC: hepatocellular carcinoma.

In HBV infection (Figure 1A), a variety of factors are dysregulated (Figure 1B), leading to immune dysfunction and liver pathogenesis, often with fatal outcomes (Figure 1C). In this review, we will further elucidate these mediators and the mechanisms behind their influence on the viral life cycle and disease progression.

## 2. Molecular Biology of HBV

With only about 3200 bases, HBV has the smallest known genome of a human DNA virus. In the infected hepatocyte, HBV exists as a small circular DNA molecule, the covalently closed circular DNA (CCCDNA), from which RNA is transcribed. These RNAs are translated into proteins or serve as the template to make new DNA, which is packaged in newly formed virus particles. HBV particles consist of an enveloped core particle, which can consist of 180 (T = 3) or 240 (T = 4) HBV core protein (C) monomers. Playing an important role in HBV diagnostics, the core protein is often referred to as the core antigen or HBcAg. In the cytoplasm, the core particle assembles around a complex formed by the viral polymerase (P) and the viral pregenomic RNA (pgRNA). Only after the formation of the viral core particle is the viral pgRNA reverse-transcribed by the P protein. Completion of the reverse transcription and the partial completion of the second (+) DNA strand lead to structural changes in the outside of the core particle and induce its envelopment. 

The viral envelope consists of a host cell-derived lipid bilayer membrane and three different envelope proteins called surface (S) proteins; the (small) S, and the S1 and S2 proteins. S and S1 are n-terminally truncated forms of the S2 proteins that are produced by alternative initiations of transcription and/or translation. When considered as a soluble antigen, the S protein is referred to as S antigen (HBsAg) in analogy to HBcAg. 

HBV entry is initiated by the binding of the preS1 domain of the HBV large surface protein to the bile acid transporter sodium taurocholate cotransporting polypeptide (NTCP) [13]. Subsequently, the HBV core particle is released into the cytoplasm and migrates to the nucleus; in this regard, the exact mode and site of release have not yet been conclusively characterized. However, it has been shown that the core particle disassembles in association with the nuclear pore and releases the partially double-stranded HBV DNA in the nucleus. Here, the viral DNA is repaired by cellular enzymes to form the fully double-stranded circular minichromosome called cccDNA. Transcription from the cccDNA is tightly regulated by the chromatin state, DNA methylation, and level and activity of transcription factors. All these regulatory mechanisms are heavily affected by extracellular signals, and as such, interaction of the HBV-infected cell with its environment is a major factor in the regulation of HBV replication. HBV expresses two nonstructural proteins: the accessory X protein (HBx), and the e antigen (HBeAg), which is a truncated form of the core protein that is secreted. HBx is essential for the initiation and maintenance of HBV RNA transcription [14]. The best-understood function of HBx is inducing the degradation of the Smc5/6 complex, which in the absence of HBx binds to the HBV cccDNA and strongly suppresses or blocks viral RNA transcription [15,16]. Besides regulating HBV RNA transcription, it has been observed that HBx expression can cause many, poorly understood effects on cells, such as the activation of cellular signalling pathways, disruption of the cell cycle, cell-cell interaction, and more. However, it is not clear how such effects may benefit viral replication, and even though they are observed in infected patients, they often do not seem to occur in other natural models of HBV infection [17], indicating that induction of cellular signalling pathways in vivo may depend on specific conditions and interactions between the infected cell and its environment. Interestingly, although the effect of HBx on HBV RNA transcription is occurring inside the infected cell, HBx expression also affects cells in a paracrine manner. For instance, HBx expression in hepatocytes induces collagen expression in HSCs in a paracrine manner [18], and can affect hepatocyte proliferation [19]. 

Compared to regulatory functions by the HBx, the functions of the HBeAg are less well understood. HBeAg is translated from the HBV core gene, when RNA transcription is initiated from an alternative transcription initiation site than that of the core RNA. This leads to translation of the core protein from an alternative 5′ in-frame start codon. This protein is differentially processed; the c- and n terminal parts are cleaved off and dimers of this truncated protein are excreted. Transcription of the e antigen is regulated by the basal core promoter (BCP) [20], and intriguingly, is often lost during or after the immune active stage of the infection. The odds of losing e antigen expression differs for the different HBV genotypes [8], and specific mutations in the basal core promoter are associated with increased risk of developing HBV infection-related HCC [21]. HBeAg expression is associated with increased viral loads, and is generally believed to contribute to immune anergy by overloading the immune system. In line with such a “strategy”, HBV-infected cells also secrete massive amounts of HBsAg, which are embedded in the membrane of rod- and cone-shaped particles. These massive amounts of excreted antigens can induce tolerance by affecting the tolerization of adaptive immune cells. On top of that, HBsAg also affects local nonparenchymal cells that may otherwise contribute to antiviral responses, such as liver sinusoidal endothelial cells (LSEC) [22]. Some reports also report the excretion of empty and HBV DNA-containing, non-enveloped HBV core particles, and large protein complexes consisting of the HBV core and e antigens, the so-called HBV Core-related antigens (HBcrAg) [23,24]. Moreover, the excretion of intact HBV core particles by HBV replicating cells has been observed in vitro [25], but if this takes place in vivo remains elusive, and whether such particles play a role in HBV replication or pathology is unclear [25]. Like the secretion of exosomes, which is the best-known group of extracellular vesicles, the secretion of HBV virions occurs via multivesicular bodies (MVBs) and depending on Alix and the ESCRT III complex, which are also essential for exosome release [26,27,28,29]. SVPs, on the other hand, are formed at the membranes of the endoplasmic reticulum, which is why their release occurs via the Golgi network [30,31,32]. This use of cellular secretion mechanisms for the release of its own gene products once again demonstrates the marked adaptation of HBV to the host and is nowadays the subject of intensive research.

## 3. Interactions between HBV Infection, Metabolism, and Hormones

Not only virally encoded proteins, but also multiple host metabolic and lifestyle factors affect HBV replication, disease progression, and pathogenesis. Several studies have shown that there is a clear relation between HBV infection and the gut microbiome. In humans, a clear correlation between gut microbiome diversity and HBV viral load can be shown [33]. However, the causative relation between the microbiome and HBV replication is very complicated, and has not yet been adequately clarified. Briefly, some studies have shown that HBV infection alters the microbiome [34,35], whereas others have shown that, conversely, the gut microbiome can affect HBV replication and disease progression. In mice, fecal transplant can significantly alter HBV replication and disease progression [36]. The increase in HCC in HBV-infected individuals is independent of other lifestyle factors that increase the odds of developing HCC, such as alcohol intake and smoking, although there seems to be a synergetic effect of low income and HBV infection on the risk of developing HCC [37]. In mice, a low-protein diet can substantially lower viral replication and the risk of developing HBV-related HCC [38]. 

HBV infection affects the host metabolism, both on the level of the infected cell and on a systemic level. Liver disease in general, whether it is related to HBV infection or not, influences the host metabolism, reduces the resting energy expenditure (REE), and causes a shift from the oxidation of glucose to fats [39]. Several studies have shown that HBV infection directly affects hepatic metabolic signalling pathways [40] and significantly affects metabolic pathways, and consequently, the excretion of metabolites such as maltotriose, maltose, myristate [14:0], arachidate [20:0], 3-hydroxybutyrate [BHBA], myo-inositol, and 2-palmitoylglycerol [16:0] [41]. Such changes appear to be largely related to the expression of the HBx protein [41]. In the further course, HBV infection-related changes in metabolism influence the interaction between hepatocytes and adaptive immune cells, and thereby affect disease progression and therapy outcome. Consequently, in HBV related HCC, a metabolism-related gene signature predicted the outcome of conventional and immune therapy [42]. 

On the subcellular level, HBV infection also has a profound effect on mitochondrial function [43], which then affects the HBV-infected cell and the liver environment in several ways. Mitochondrial dysfunction causes oxidative stress, which can lead to the formation of radical oxygen species (ROS), which can cause DNA damage and may thereby contribute to HBV infection-related carcinogenesis. Notably, ROS species diffused out of stressed cells also affect other nearby cells than the cell where they are produced, and may cause DNA damage and cellular stress in uninfected bystander cells. The cellular stress leads to changes in proliferation, cell death, and survival, and thereby contributes to fibrosis and cirrhosis [43,44].

As a major hepatotrophic virus, HBV infection also alters bile acid (BA) metabolism in several ways. The disruption of BA uptake by direct interaction between HBsAg and the NTCP [45], which is the major hepatic bile acid transporter, can lead to cholestasis, a disease characterized by high serum levels of bile acids. In humanized mice, both HBV infection and administration of Myrcludex-B—a competitive HBV/HDV entry inhibitor consisting of a portion of HBV surface antigen that binds to and disrupts the function of NTCP—strongly induced cholesterol 7α-hydroxylase (CYP7A1), the rate-limiting enzyme for the conversion of cholesterol to bile acids [46]. Clinical trials for the application of Myrcludex-B to treat hepatitis D virus (HDV) infection in humans have shown that Myrcludex-B administration leads to increased plasma bile acid levels in HBV-infected patients [46], although this does not seem to lead to any symptoms or adverse effects. In a mouse model of HBV infection, the HBV infection-related cholestasis caused a decrease in CD25+/CD69+ CD4+ and CD8+ cells, while CTLA-4+ CD4+ and CD8+ subsets were increased. Thus, HBV infection-related cholestasis may contribute to immune dysfunction [47].

Since the liver plays an important role in the regulation of lipid metabolism, HBV infection unsurprisingly leads to altered lipid synthesis [48]. It has been demonstrated that HBV infections directly affect the expression of genes involved in lipid biosynthesis [49]. Furthermore, HBV infection has systemic effects on lipid metabolism. In this way, HBV infection directly affects the production of Apolipoprotein A1 (ApoA1) [50] by hepatocytes. This protein is a major component of lipoprotein complexes that transport lipids and cholesterol between the liver and other systemic cells. The deregulation of lipid metabolism by HBV infection may also lead to steatosis, especially in patients with other metabolic comorbidities, such as insulin resistance [51]. Interestingly, meta-analysis indicates that the risk of developing NAFLD is significantly lower in CHB patients [52].

It has been observed that the host sex has a major effect on the disease progression and the development of HBV infection-related pathogenesis. Men are 2.08 times more likely to develop severe liver disease than women [53,54]. Both hormones and sex-related differences in immune function affect HBV replication and HBV infection-related pathology. It has been shown that the female hormone estrogen suppresses HBV replication, at least partially by reducing Hepatocyte Nuclear Factor 4 Alpha (HNF4α) activity [55]. In this context, sex-specific cellular microRNA (miRNA) levels are also important, as in humans with HBV-related cirrhosis, miRNA expression is altered in a sex-specific manner [56]. Conversely, no major effects of HBV replication on host (sex) hormones have been described.

## 4. HBV and the Immune System

### 4.1. Sensing of HBV Infection by the Innate Immune System

**Figure 2:**
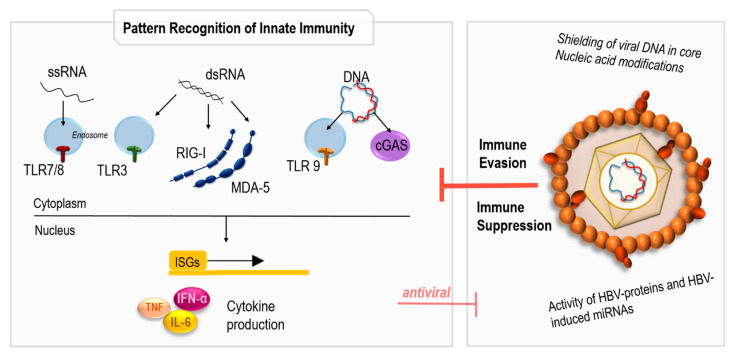
Pattern recognition of HBV infection. Intracellular pathogenic nucleic acids are recognized by endosomal Toll-like receptors (TLR) and cytosolic retinoic acid inducible gene I (RIG-I), melanoma differentiation antigen 5 (MDA-5) or cyclic GMP-AMP synthase (cGas), leading to cytokine production and transcription of interferon-stimulated genes (ISGs). Hepatitis B virus (HBV) is capable of both evading and suppressing pattern recognition of innate immunity. dsRNA: double-stranded RNA; IFN-α: interferon α; IL-6: interleukin 6; miRNA: microRNA; ssRNA: single-stranded RNA; tumour necrosis factor.

The innate immune system represents the evolutionarily older form of immune response and is activated when genetically encoded pattern recognition receptors (PRRs) recognize a danger signal [57,58]. This danger signal may be, for example, a DNA or RNA that is recognized as a pathogen-associated molecular pattern (PAMP) because it is not present in that particular cellular compartment or with that structure under physiological conditions [59,60,61]. Furthermore, certain nucleic acid compositions or modifications, such as the absence of immunoinhibitory methylations, may be characteristic for a PAMP and induce PRR activation [62]. Regarding the recognition of potentially pathogenic RNA, two families of PRRs are responsible in human cells: Within the endosome, single-stranded RNA (ssRNA)-detecting Toll-Like Receptors (TLRs) 7 and 8 and double-stranded RNA (dsRNA)-detecting TLR3 are localized, evoking the immune response to pathogen infection [59] (Figure 2). In the cytoplasm, dsRNA structures are detected by Retinoic acid inducible gene-I (RIG-I) and melanoma differentiation-associated protein 5 (MDA5), which belong to the family of RIG-I-like receptors (RLRs) [63]. Regarding DNA recognition, TLR9 detects DNA ligands with unmethylated CpG-motifs in the endosome [64], whereas cytoplasmatic DNA is detected via the cGAMP synthase–stimulator of interferon genes pathway (cGAS-Sting pathway) [65]. Regardless of their nature and localization, all of these PRRs have in common that upon binding of their specific PAMPs, they activate cellular signalling pathways that induce a cytokine response and transcription of interferon-stimulated genes (ISGs) which inhibit viral replication [57]. Therefore, recognition of viral infection by the innate immune system is a severe disadvantage for the virus, which is why viruses have evolved mechanisms to evade or inhibit PRR sensing, and thus promote the spread of their own genetic information [57]. 

It is not surprising that HBV, as a virus optimally adapted to the human immune system over the course of its long coevolution, is generally believed to replicate without activating an innate immune response. Thus, HBV is often considered a “stealth” virus [66,67,68]. This ability of HBV to circumvent detection by the innate immune system seems to involve both immunoevasive and immunosuppressive components: One important aspect of HBV immune evasion is that viral replication has evolved such that viral nucleic acids are shielded from PRRs and thus are not recognized. For instance, viral DNA is produced in the viral core particle, preventing binding of the viral DNA by immune sensors [66]. Likewise, immune recognition of viral RNA is at least partially inhibited: Although it has been reported that the epsilon region of the pregenomic RNA of HBV is recognized by RIG-I, inducing type III interferon expression [69], it is also known that RIG-I activation is prevented by N6-methyladenosine modification of the epsilon structure [70]. In addition to efficient immune evasion, the virus seems to actively suppress different signalling pathways that are activated in response to innate sensing of a viral infection. Most of these effects are the result of direct interactions of the viral proteins, and of HBV-induced miRNAs [71,72,73]. For example, direct immunosuppression by HbsAg itself, which is the main component of HBV-SVPs, has been reported [74,75]. Taken together, the interaction of HBV with PRRs of the innate immune system is a highly complex topic that has been extensively discussed but still requires further research (see also [72,73,76,77] for review).

### 4.2. Immune Response to HBV Infection

The interactions between the HBV-infected cell and the adaptive immune system have a major influence on the course of HBV infection. In people infected as adults, a strong adaptive immune response clears the virus in about 90% of cases. Conversely, in young children, and especially in perinatally infected children, the infection becomes chronic in about 90% of cases [3]. To persist in chronic infection, HBV affects the host immune system in various ways. Very characteristic for hepatitis B virus infection are the massive amounts of viral proteins (HBsAg and HBeAg) that are excreted. 

These proteins are thought to “overload” the immune system in a manner that leads to tolerance to the antigens [78,79]. Especially in individuals infected perinatally or in early childhood, this tolerance is so extended that no immune responses against the virus can be detected. This phase of the infection is therefore called the immune-tolerant phase, in which the amount of virus in the blood is typically very high and can reach levels of 106-107 IU/ml HBV particles. Despite this high viral load, ALT and AST levels are normal, indicating that little to no damage to the liver occurs during this stage. 

Immune responses develop in most HBV-infected individuals at some point, often after decades of immune tolerance. As their immune cells are typically partially functional, their antiviral activity commonly leads to a “status quo”, in which the recognition of HBV-infected cells results in the release of cytokines that cause inflammation and damage the liver, but do not clear the infection [4]. This immune dysfunction is characterized by dysfunctional HBV-specific T cells that have an “exhausted” phenotype [80,81].

In their fully functional and non-exhausted shape, virus-specific CD8+—and to some extent, CD4+ T cells—can release mediators such as cytokines and perforin/granzymes (CD8+ T cells) that suppress viral replication, destroy the viral cccDNA, or kill the infected cell. Interferon-α (IFN-α), a major cytokine released by a multitude of cell types, suppresses HBV RNA transcription and can induce cccDNA degradation [82,83]. Because of this, IFN-α has been used in the past to treat HBV infection, leading to sustained viral responses (lower viral load) in about half the patients, when treated for about 6-12 months [84]. However, IFN-α therapy is currently rarely used due to its heavy side effects and its limited clinical benefit in only a subset of patients. In resource-rich settings, CHB is mostly treated with nucleos(t)ide analogues (NAs) that directly suppress viral replication by blocking the conversion of pgRNA in viral DNA and have few side effects [84].

It has been shown that in HBV infection, there are multiple interactions between viral activity and cytokines secreted by hepatocytes, other cells of the liver parenchyma, and cells of the innate and adaptive immune systems. Many hormones and cytokines affect HBV replication, and conversely, HBV replication directly affects the systemic and local levels of cytokines, which has profound effects on systemic and intrahepatic processes in the HBV-infected host. For instance, it has been shown that HBV replication is profoundly suppressed by the secretion of various interleukins (IL) such as IL-6 and IL-18 [85], IL-21 [86], or IL-33 [87]. Consequently, polymorphisms in cytokine genes that affect their expression may affect the disease progression in humans [88,89] and the odds of developing HBV infection-related HCC [90]. 

Tumour Necrosis Factor Alpha (TNF-α) also strongly suppressed HBV replication in a mouse model that systemically applied interleukin-2 suppressed HBV replication in an TNF-α dependent manner [91].

HBV infection affects serum levels of various cytokines that affect other cells on a local and systemic level. HBV infection causes an increase in serum IL-35 [92], which has been demonstrated to induce immune tolerance. IL-35 can increase the production of IL-10 and IL-35 itself in regulatory T cells, but also affects the activity of effector T cells by reducing IFN-γ and TNF-α production [92]. Moreover, IL-6 is increased in CHB, and may have profound effects on HBV disease progression (reviewed in [93]). Exerting a multiple of regulatory functions, IL-6 can directly affect immune cells and induce a regulatory phenotype, but it may also directly affect the expression and activity of proteins involved in absorption, distribution, metabolism, and excretion in liver cells.

Furthermore, noninfected liver parenchymal cells, such as Kupffer cells and liver sinusoidal endothelial cells (LSEC), are directly affected by HBV infection, and play a role in the dynamics of the local cytokine environment. It has been demonstrated that exposure to HBV-infected cell supernatants can suppress TLR signalling in several types of parenchymal liver cells [74]. This may benefit HBV persistence by interfering with the recognition of HBV virions by nonparenchymal liver cells. It has been described that the recognition of HBV virions by nonparenchymal liver cells can suppress HBV replication by inducing the release of proinflammatory cytokines IL-6, IL-8, TNF-α, and IL-1β [94]. In line with this observation, it was recently shown that HBV replication can revert the production of proinflammatory cytokines to anti-inflammatory. 

### 4.3. Role of Innate and Adaptive Immune Response and Related Cytokines on HBV-Related Carcinogenesis

It has been estimated that untreated HBV infection leads to HCC in 10–25% of cases. Many aspects of CHB contribute to the development of HCC. In immune active CHB, the ongoing immune responses cause inflammation, which can lead to fibrosis and later to cirrhosis. In particular, CHB-related cirrhosis makes the liver prone to developing HCC, and most CHB-related HCC develops in this setting. Notably, this is also the case for HCC of other aetiologies, such as alcohol overconsumption or inflammation due to hepatitis C virus (HCV) infection. A hallmark of liver fibrosis and cirrhosis is the “scarification” of the liver tissue, the replacement of healthy liver cells by increasing amounts of collagen. It has been demonstrated that collagen excretion by hepatocytes is increased upon exposure to cytokines [95] and growth factors, such as TGF-β. Cytokines also drive another key component of HCC; the activation of signalling pathways, such as MAPK/ERK and JAK/STAT3. 

The activation of these pathways begins with the binding of signalling proteins, such as cytokines, to cell surface receptors. Subsequently, the ‘second messenger’ proteins, e.g., STAT3 and ERK, are activated by phosphorylation, upon which they transfer to the cell nucleus, where they bind responsive elements in promoters to regulate processes such as proliferation and survival. Under physiologic conditions, STAT3 signalling is transient because typically the STAT3-inducing cytokines are transiently produced, for instance, by T cells or by stressed or damaged hepatocytes, and because of feedback mechanisms that dephosphorylate the protein and translocate it back to the cytoplasm. Prolonged activation of the STAT3 signalling pathway mostly induces cell death, but under some conditions it can activate a feedback loop, in which STAT3 induces the cytokine IL-6, a powerful activator of STAT3 signalling. In many HCCs, if not all HCCs, the feedback mechanisms that regulate STAT3 activity are dysfunctional, leading to a poorly understood pattern of aberrant, constitutive, and potentially self-amplifying activation of STAT3 signalling. This constitutive STAT3 signalling is crucial for the survival of HCC, and it is possible to selectively kill HCC cells by interfering with STAT3 signalling [96]. This makes STAT3 signalling an interesting therapeutic target, especially because it is not essential for the functioning of normal hepatocytes. For instance, Tyrosine kinase inhibitors (TKIs) are applied, which kill HCC cells by blocking the proteins that phosphorylate second messengers, such as ERK and STAT3. In particular, the TKIs sorafenib, lenvatinib, cabozantinib, and regorafenib should be mentioned here, which are successfully used for the treatment of HCC.

Their efficacy underlines the importance of cell-to-cell signalling events, not only in the induction, but also in the survival of HCC cells. HBV replication activates the ERK and STAT3 signalling pathways in a paracrine and HBx-dependent fashion [97], but the mechanism and involvement of cytokine(s) is largely incompletely understood [98]. Presumably, HBx-induced IL-6 is partially responsible for the paracrine effects of HBx [99], but other cytokines may be involved as well [98,100].

The intimate relation between cytokine secretion, cytokine signalling, and HCC points out the complexity of the interactions between the HBV-infected cell and its environment, and the importance to CHB-related pathology. Thus, these interactions may explain the intimate relation between (aberrant) innate and adaptive immune activation, inflammation, and HCC. It has been demonstrated that IL-6 is increased in people with CHB and in people with HCC. IL-6 can be produced by regulatory CD4+ T cells, but also by hepatocytes. Hepatocytes are principally capable of inducing other immunosuppressive cytokines as well, such as IL-10 and TGF-beta [100,101]. Besides stimulating ERK/STAT3 pathway activation, these cytokines also affect multiple cell populations in the liver, leading, amongst others, to HSC-mediated collagen deposition, thereby contributing to liver fibrosis, which often precedes HBV-related HCC [102]. There are currently no therapeutics that directly target the cytokines that are involved in HCC induction and survival. 

It is likely that the most direct driver of aberrant cytokine and growth factor production in the HBV-infected liver is the HBx protein. HBx expression affects cells in a paracrine manner, and can affect processes such as collagen excretion [18], proliferation [19]. The HBx-induced cell-cell interactions are poorly understood. In particular, which cytokines are induced—and under what conditions—is a matter of debate.

## 5. Extracellular Vesicles in HBV Infection

### 5.1. Extracellular Vesicles in Viral Infection

Extracellular vesicles (EVs) are small membrane-enveloped vesicles that are actively released by a wide variety of cell types and contain information about the state of the cell at the time of its biogenesis [29]. This information is characterized by a distinct EV cargo (e.g., proteins, microRNAs, or other nucleic acids) that is transported from a cell of origin to a target cell [103,104]. Therefore, EVs are considered as important signalling vehicles and modulators of cellular functions such as the immune response. According to their mode of biogenesis, EVs are classified into different subgroups. The most prominent subgroups are exosomes, which are generated by inward budding into the multivesicular bodies (MVBs); microvesicles, which are released by outward budding of the plasmamembrane; and apoptotic bodies, whose formation is associated with cell death. In the following, the generic term “extracellular vesicles” will be used, since a separation and classification of already-released EVs into the individual subspecies is not possible with conventional purification methods [105]. These methods purify EVs according to their density (gradient centrifugation), diameter (size-exclusion chromatography) or on the basis of specific surface antigens (affinity purification), resulting in EVs of relatively high purity. Moreover, EVs can be purified based on their sedimentation speed (ultracentrifugation) or precipitated using reagents such as polyethylene glycol; these two methods isolate EVs of lower purity. However, the removal of cellular contamination during EV purification is not the only difficulty in immunological EV research. Due to the marked similarities between enveloped viruses and EVs in their size, density, and membrane composition, it is also not straightforward to separate them [106,107,108]. Despite all these controversies and difficulties, encapsidation of infectious viral genomes of whole enveloped and non-enveloped viruses has been reported by several groups to occur and contribute to disease progression [109,110,111,112,113,114]. This route of dissemination is of particular immunological interest and especially advantageous for the virus, as it is protected in this way from both enzymatic degradation and neutralization by antibodies [111,115,116,117]. Still, the role of EVs in infection biology goes far beyond EV-induced viral spread: even if they do not contain viral genomes, virus-induced EVs can act directly on the innate immune response, both activating and inhibiting its immune cells and signalling pathways [116,118,119]. Since this also occurs in the case of HBV infection, the present findings will be described in more detail in the following chapters.

### 5.2. Immunomodulatory EVs in HBV Infection

**Figure 3:**
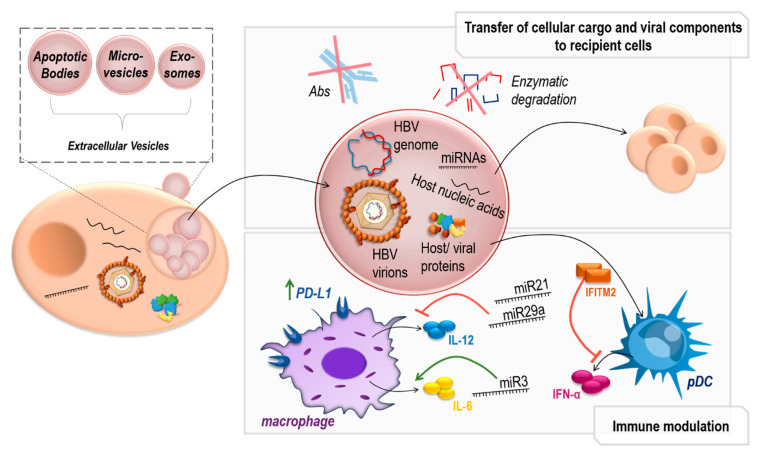
Roles of extracellular vesicles in hepatitis B virus infection. Extracellular vesicles (EVs) are released from the plasma membrane or multivesicular bodies and contain specific cargo such as proteins, nucleic acids, and microRNAs (miRNAs), which they transport to recipient cells and protect from enzymatic degradation. In hepatitis B virus (HBV) infection, HBV proteins, genomes, and virions are also incorporated into EVs and thus are shielded from neutralizing antibodies. HBV-induced EVs inhibit the immune response by shuttling interferon-induced transmembrane protein 2 (IFITM2) to plasmacytoid dendritic cells (pDC) blocking interferon-α (IFN-α) release and by inducing miR21 and miR29a, which downregulate interleukine (IL)-12 production (indicated with red arrow). Upregulation of programmed cell death ligand 1 (PD-L1) or interleukin 6 (IL-6) is indicated with green arrow.

Since HBV, as a virus optimally adapted to the human host, skillfully manipulates the immune response, it is hardly surprising that it also uses EVs for this purpose (Figure 3). In recent years, several valuable studies have been published describing an impact of EVs on HBV infection. However, the authors of these publications likewise faced the difficulty of adequately separating EVs and virions, as these are highly similar in size, density, and envelope composition [107,108]. Consequently, the extent to which EV-induced immunomodulation can be addressed by the respective experimental setups must be critically considered.

A particular mechanism of HBV-induced immunomodulation is associated with subviral particles (SVPs) such as filaments and spheres, which can be considered as a specific subset of EVs untypically released from the endoplasmic reticulum via the Golgi network [30,31,32]. However, since these SVPs also contain viral components such as HbsAg [120], they can be considered as an intermediate between EVs and virions [108]. This is reinforced by reports from Jiang et al. according to which SVPs, like HBV virions and exosomes, originate from multivesicular bodies, further emphasizing the relatedness between these different extracellular entities [121]. Although the data regarding the immunomodulatory role of SVPs are sparse, influences on both the adaptive and innate immune systems are becoming apparent. Not only have SVPs been shown to sequester neutralizing antibodies in patient sera [120], but an inhibitory effect of SVPs on interferon-α (IFN-α) release by human plasmacytoid dendritic cells (pDCs) has also been reported [75]. As the authors also observed inhibition of IFN-α release by treatment with recombinant HbsAg derived from yeast, the question arises as to whether immunosuppression by SVPs is due to their HBsAg content only, or also due to other EV cargo such as virus-induced miRNAs or other viral proteins [74,75]. These interesting observations call for further studies on the immunomodulatory role of SVPs.

In addition, immunosuppressive effects of SVP-free EVs (non-SVP EVs) have been reported at the functional level. It was observed that EVs which were purified from the supernatants of HBV-producing cells, as well as from patient sera accumulated in liver, spleen, and intestine [122]. This was concluded to induce immune suppression and enhanced numbers of HBV core antigen-producing cells in a murine in vivo system. However, EVs were purified by ultracentrifugation, resulting in rather impure EVs and no separation between EVs and HBV virions. In another study, EVs were separated from SVPs and HBV virions by density gradient centrifugation, and a separation between fractions containing EV-marker CD9 on the one hand and fractions containing HbcAg and HBV DNA on the other hand was shown [123]. These EVs were then used to tread human peripheral blood mononuclear cells (PBMCs), and upregulation of programmed death ligand 1 (PD-L1) in macrophages and monocytes was measured. The authors suggest that this may be responsible for the frequent occurrence of T-cell exhaustion in CHB patients [123,124]. In line with this, a study by Shi et al. concludes that HBV-induced EVs are also partly responsible for the decreased response of some CHB patients to IFN-α treatment. The authors claim that interferon-induced transmembrane protein 2 (IFITM2), which is upregulated in CHB, will be shuttled to pDCs by exosomes and thus inhibit IFN-α synthesis [125]. However, EVs in this study were purified by precipitation, resulting in a decrease in the volume of an EV sample rather than removal of non-EV components. Thus, no distinction can be made at this point between effects due to exosomes, other EVs, and other components in the conditioned medium because all are precipitated.

After all this evidence for an immunomodulatory role of EVs in HBV infection, the question arises as to which viral or virus-induced factors are responsible for these effects. The HBx protein, as the master regulator of HBV infection, also seems to play an important role in this context. Thus, HBx induces the release of EVs that transfer both HBx protein and mRNA to hepatic recipient cells, which stimulates them to proliferate, and thus may be partly responsible for the formation of HCC [126]. Moreover, HBx has been reported to enhance the EV-dependent secretion of the HBV replication-inhibiting apolipoprotein B mRNA-editing catalytic polypeptide 3G (APOBEC3G), lowering intracellular APOBEC3G levels and thus promoting HBV replication [127]. 

However, HBx is not the only immunoregulatory factor in HBV infection. For instance, cellular and viral microRNAs (miRNAs) can be transported by EVs, thus affecting the immune response to HBV infection. Two reports by the group of Hua Tang published in 2017 and 2020 address the HBV-encoded miRNA miR3, which promotes HBV persistence and controls viral replication [128,129]. They observed that miR3 is found in EVs as well as in the HBV virion itself, enhances IL-6 secretion via SOCS5 in M1 macrophages, and thus controls the innate immune response. In addition to these reports of immune regulation by HBV-encoded miRNAs, there are further reports that HBV also affects the expression of cellular miRNAs. Kouwaki et al. demonstrated that HBV infection leads to increased levels of cell-derived immunoregulatory miR21 and miR29a in EVs, thereby inhibiting the IL-12 response in THP-1 macrophages [103]. In this way, HBV can not only target EV pathways but also influences cellular miRNA biogenesis, thus linking two completely different regulatory pathways and using them for its own advantage.

Like all areas of EV research, the focus of HBV-induced EVs is in an evolving process, and benefits greatly from new methods. Previously mentioned studies did not yet have access to an established method allowing for complete removal of HBV virions from EV samples, which is essential for a final differentiation between virus- and EV-mediated effects. The use of EVs in functional studies also requires that the samples are not contaminated with antibodies. However, EV samples obtained by affinity-based methods still contain EV-specific antibodies, which cannot be separated from the vesicles if they have been purified by positive selection. Therefore, purification of pure EV samples and removal of HBV contamination by negative selection is the method of choice. In 2020, we published a method showing a clear removal of HBV virions from EV-containing plasma samples [130]. This method is a combination of size exclusion chromatography and removal of HbsAg-positive particles by negative selection. The resulting EV samples are free of HBV-sized particles, HbsAg, HbcAg, and infectious potential. As the samples are also free of contaminating HbsAg-targeting antibody, they are suitable for functional studies. These new methodologies enable a clear distinction between virion and EV-mediated effects.

Regarding the many observations on proviral and immunosuppressive EVs in HBV infection, it should not be forgotten that HBV-induced EVs may also play an immune-activating role. However, the nature of PRR-activating ligands could not be conclusively identified in this context. Indeed, EVs released from HBV-infected hepatocytes were reported to induce upregulation of NKG2D ligand on THP-1 and Hepatic F8/80+ macrophages, which resulted in increased IFN-γ secretion from NK cells when cocultured [103]. However, immune-activating EVs were suspected to contain ligands for both RLRs and TLRs, but the immune-activating nucleic acids were not identified.

In another study, Dansako et al. primarily suspected immune activation through EV-dependent transfer of mitochondrial DNA [131]. However, it should be noted that this specific observation may be due to apoptotic bodies as a particular subset of EVs. One reason why this conclusion should be cautiously considered is that EV purification was performed via ultracentrifugation, which does not yield EVs of the highest purity, and no filtering step to remove apoptotic bodies was applied. The other reason is that although HBV infection did not lead to less cell viability, it did lead to a reduction in cell number.

Compared to the publications of EVs in HBV infection, the number of publications on immunomodulatory EVs in HDV infection, which is a satellite virus dependent on HBV, is much lower. In the first publication ever to establish a link between EVs and HDV, we recently showed that HDV monoinfection, which does not induce virus release in the absence of HBV, mediates the release of immune-activating EVs [132]. These EVs induced a proinflammatory cytokine response in noninfected primary human immune cells. Another publication reports that EVs artificially loaded with HDAg can elicit a cellular immune response against HDV, but the role of HDV-induced EVs in HDV infection was not examined in this study [133]. Apart from these two publications mentioned above, we are not aware of any other original publications on the role of EVs in HDV infections. The various indications of immunomodulatory effects of EVs in HBV and HDV infections call for further research in this area.

### 5.3. Transfer of HBV Genomes in HBV-EVs

EVs not only influence disease courses by immune manipulation, but also via direct transfer of viral genomes, and this has also been reported for HBV-induced EVs. This way, EV-dependent transfer of replication competent HBV genomes could lead to antibody-independent viral spread, enhancing the severity of hepatitis virus disease. Given the challenging nature of separating EVs from HBV virions and the lack of an established method for EV-HBV separation at the time the experiments were conducted, these individual studies require detailed examination and in-depth discussion:

Yang et al. reported that EVs purified from the sera of chronic hepatitis B virus carriers contain viral nucleic acids, which mediate viral transmission [134]. What suggests a high purity of these EV samples is that they were obtained by CD63-specific affinity isolation, which is considered a method for purifying low-contaminant EVs. However, postulated EV samples still contained HbsAg, and an absence of hepatitis B virions was not shown. Additionally, it cannot be excluded that CD63 is also associated with infectious HBV particles, as it is necessary for the assembly of HBV in the multivesicular body [121,135]. In line with the previously mentioned publication, Kouwaki et al. also detected HBV RNA in EVs released from NTCP-expressing and HBV-infected HepG2 or Huh7 cells [103]. A CD81-specific positive selection was applied to purify the EVs, via which very pure EVs can also be obtained, though it is also not clear if CD81 is excluded from the HBV virion.

In contrast, Kakizaki et al. suggest that HBV DNA copies are more likely to be present in the HbsAg- and HBcAg-containing virion fractions of a density gradient than in the EV-containing fractions [123]. In this context, it is important to note that EVs occur in a variety of subspecies that also differ in density, so the putative HBV viral fraction may also contain EVs [136,137].

In a recent study, Wu et al. reported enclosure of full HBV virions inside EVs and demonstrated it by electron microscopy [138]. So far, this effect has been predominantly reported for naked viruses such as hepatitis A and E virus, and future work is required to show whether these EV-cloaked virions are specifically translocated into EVs or end up in apoptotic bodies through cell death [110,111,112,113,114,139].

According to further publications, HBV DNA was detected in EVs from patient plasma as well as in EVs released from HBV-infected primary hepatocytes or cell lines [131,140,141]. However, these studies employed ultracentrifugation for EV purification, which does not separate EVs from virions. Consequently, they might indicate EV-mediated spread in HBV infection, but may not be sufficient to prove it, as effects could also be due to residual infectious potential of contaminating virions.

In agreement with the results of other research groups, we also detected HBV genomes in EVs after complete HBV virion removal [130]. Since these EV samples were free of HBsAg and HBcAg, the HBV genomes could only be present in EVs or in naked capsids not enveloped by HBsAg [142]. Regarding HDV as the satellite virus to HBV, HDV genome-containing EVs were also present in the conditioned medium of HDV-monoinfected hepatoma cells releasing only EVs and no HDV virions due to the lack of HBsAg [132].

Taken together, these combined results shown by various groups demonstrate that encapsidation of HBV genomes occurs in EVs and may have an impact on infection progression. If the postulated EV-dependent HBV transmission is shown to be a fact in further studies, it would have a striking impact on the course of infection and the efficacy of therapies. Given the high number of chronically infected patients, further research in this area is absolutely essential.

## 6. Conclusions

Given the broad spectrum of systemic and intrahepatic processes affected by HBV infection, it is crucial to understand the complex interplay between HBV-infected cells and their environment. This includes direct cellular interactions between HBV-infected and noninfected liver or immune cells, but also a variety of mediators that are essentially involved in the complex interaction between HBV infection and its associated pathology. These interactions are currently being studied in detail to determine whether the observed effects are directly caused by the virus or are due to the action of such mediators. With 820,000 annual deaths associated with chronic hepatitis B, a profound understanding of these influencing factors on disease progression, HBV-related carcinogenesis, and therapeutic outcomes is urgently needed.

## Data Availability

Not applicable.
